# Activation of cytosolic phospholipase A_2 _in dorsal root ganglion neurons by Ca^2+^/calmodulin-dependent protein kinase II after peripheral nerve injury

**DOI:** 10.1186/1744-8069-5-22

**Published:** 2009-05-02

**Authors:** Shigeo Hasegawa, Yuta Kohro, Makoto Tsuda, Kazuhide Inoue

**Affiliations:** 1 Department of Molecular and System Pharmacology, Graduate School of Pharmaceutical Sciences, Kyushu University, 3-1-1 Maidashi, Higashi-ku, Fukuoka 812-8582, Japan

## Abstract

**Background:**

Peripheral nerve injury leads to a persistent neuropathic pain state in which innocuous stimulation elicits pain behavior (tactile allodynia), but the underlying mechanisms have remained largely unknown. We have previously shown that spinal nerve injury induces the activation of cytosolic phospholipase A_2 _(cPLA_2_) in injured dorsal root ganglion (DRG) neurons that contribute to tactile allodynia. However, little is known about the signaling pathway that activates cPLA_2 _after nerve injury. In the present study, we sought to determine the mechanisms underlying cPLA_2 _activation in injured DRG neurons in an animal model of neuropathic pain, focusing on mitogen-activated protein kinases (MAPKs) and Ca^2+^/calmodulin-dependent protein kinase II (CaMKII).

**Results:**

Pharmacological inhibition of either p38 or extracellular signal-regulated kinase (ERK) in the injured DRG, which led to suppression of the development of tactile allodynia, did not affect cPLA_2 _phosphorylation and translocation after nerve injury. By contrast, a CaMKII inhibitor prevented the development and expression of nerve injury-induced tactile allodynia and reduced both the level of cPLA_2 _phosphorylation and the number of DRG neurons showing translocated cPLA_2 _in response to nerve injury. Applying ATP to cultured DRG neurons increased the level of both phosphorylated cPLA_2 _and CaMKII in the vicinity of the plasma membrane and caused physical association of these two proteins. In addition, ATP-stimulated cPLA_2 _and CaMKII phosphorylation were inhibited by both a selective P2X_3_R/P2X_2+3_R antagonist and a nonselective voltage-dependent Ca^2+ ^channel (VDCC) blocker.

**Conclusion:**

These results suggest that CaMKII, but not MAPKs, has an important role in cPLA_2 _activation following peripheral nerve injury, probably through P2X_3_R/P2X_2+3_R and VDCCs in primary afferent neurons.

## Background

Peripheral nerve injury leads to a persistent neuropathic pain state in which innocuous stimulation elicits pain behavior (tactile allodynia). Effective therapy for this pain is lacking, and the underlying mechanisms have remained largely unknown. We have previously shown that spinal nerve injury induces the activation of cytosolic phospholipase A_2 _(cPLA_2_), a Ca^2+^-dependent subclass of the PLA_2 _family [1], in DRG neurons, and that inhibiting cPLA_2 _suppresses nerve injury-induced tactile allodynia, revealing a crucial role for this enzyme in neuropathic pain [2]. Activated cPLA_2 _hydrolyzes the sn-2 position of glycerophospholipids to release arachidonic acid and lysophospholipid, and subsequently generates lipid mediators such as prostaglandins, leukotrienes, platelet-activating factor and lysophosphatidic acid. These mediators have been reported to cause sensitization of primary afferent neurons [3-5] and to produce allodynic behaviors [6-9]. Activation of P2X_3 _and P2X_2+3 _receptors (P2X_3_R/P2X_2+3_R), ionotropic ATP receptor subtypes, is involved in nerve injury-induced cPLA_2 _activation in DRG neurons [2]; however, the mechanism underlying cPLA_2 _activation via P2X_3_R/P2X_2+3_R remains to be elucidated.

The activation of cPLA_2 _is regulated by phosphorylation of serine residues in addition to a rise in intracellular Ca^2+ ^concentration [10]. The catalytic domain of cPLA_2 _contains several phosphorylation sites, Ser505, Ser515 and Ser727, which have been reported to be phosphorylated by mitogen-activated protein kinases (MAPKs) [11-13], Ca^2+^/calmodulin-dependent protein kinase II (CaMKII) [14] and MAPK-interacting kinase 1 (MNK1) or a closely related isoform [15], respectively. Among these serine residues, phosphorylation of cPLA_2 _at Ser505 and Ser727 has been shown to be important for agonist-induced arachidonic acid release in mammalian cell models [11,15-17]. It is possible that the phosphorylation of these three serine residues may be interactive, because MNK1 is activated by MAPKs such as p38 and extracellular signal-regulated kinase (ERK) [18], and CaMKII modulates ERK activation [19,20]. Indeed, it has been recently shown that phosphorylation on Ser505 by ERK is dependent upon Ser515 phosphorylation via the activation of CaMKII in vascular smooth muscle cells [21].

Among protein kinases involved in cPLA_2 _activation described above, MAPKs and CaMKII are expressed in DRG neurons and have important roles in pain signaling. Nerve injury induces an increase in p38 and ERK phosphorylation in DRG neurons and injection of these inhibitors attenuates nerve injury-induced tactile allodynia [22], strongly suggesting that MAPK activation in primary afferent neurons participates in neuropathic pain after nerve injury. CaMKII, which is especially abundant in the nervous system, has been implicated in various neuronal functions, such as the synthesis and release of neurotransmitter, modulation of ion channels and receptors, gene expression and synaptic plasticity. Recently, it was reported that CaMKII is localized in small- and medium-diameter DRG neurons that are known to transmit nociceptive signals [23,24]. Intraplantar injection of complete Freund's adjuvant (CFA), a model of inflammatory pain, increases the expression of CaMKII in sensory neurons [24], and CaMKII activation regulates the activity of transient receptor potential vanilloid type 1 (TRPV1) [25,26]. Thus, it raises the possibility that peripheral nerve injury may induce the activation of cPLA_2 _via the phosphorylation of MAPKs and CaMKII in primary afferent neurons, but their roles remain to be determined.

While cPLA_2 _is distributed throughout the cytoplasm in the normal condition, in response to a variety of extracellular stimuli, an increase in intracellular Ca^2+ ^concentration promotes binding of Ca^2+ ^to the C2 domain and then allows cPLA_2 _to translocate to the perinuclear region, including the nuclear envelope, Golgi apparatus and endoplasmic reticulum in non-neuronal cells [27-31]. By contrast, our previous study showed that phosphorylated cPLA_2 _translocates to the plasma membranes of injured DRG neurons. Therefore, the translocation of cPLA_2 _in DRG neurons seems to be unique, but the mechanism of cPLA_2 _translocation remains unknown. In the present study, we investigated the involvement of MAPKs and CaMKII in cPLA_2 _phosphorylation and translocation in DRG neurons following peripheral nerve injury using pharmacological and molecular approaches.

## Results

### Inhibition of neither p38 nor ERK prevents the activation of cPLA_2 _after nerve injury

**Figure 1:**
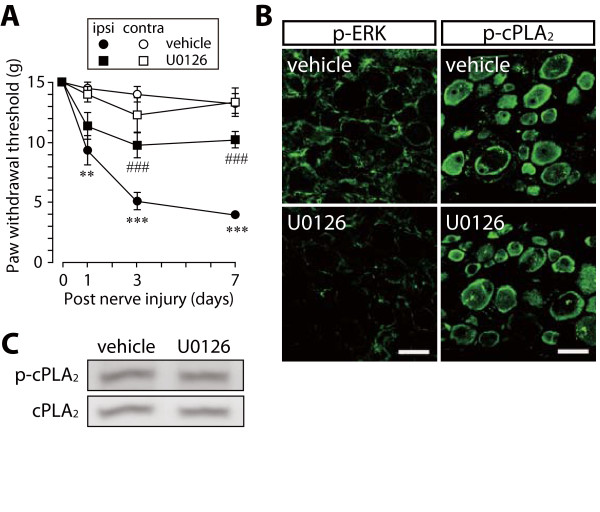
**A MEK inhibitor U0126 does not prevent cPLA_2 _activation after nerve injury**. (A) Effect of U0126 on the development of nerve injury-induced tactile allodynia. Results are means ± SEM of the paw withdrawal thresholds on the ipsilateral (ipsi) and contralateral (contra) sides. **p < 0.01, ***p < 0.001 compared with the threshold on day 0. ###*p *< 0.001 compared with the threshold of the vehicle-treated group. (B, C) Immunohistochemical (B) and western blot (C) analyses of p-ERK and p-cPLA_2 _proteins in the ipsilateral L5 DRG of vehicle- and U0126-treated rats 7 days after nerve injury. Scale bar, 50 μm.

**Figure 2:**
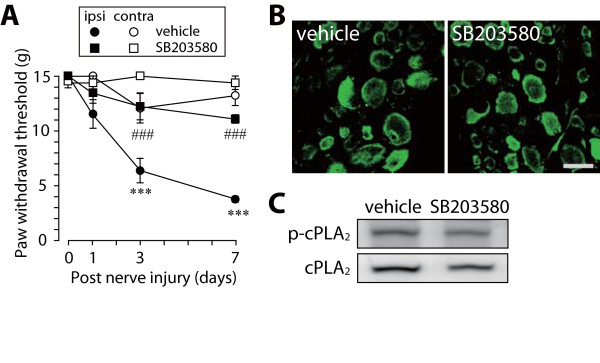
**A p38 inhibitor SB203580 does not prevent cPLA_2 _activation after nerve injury**. (A) Effect of SB203580 on the development of nerve injury-induced tactile allodynia. Results are means ± SEM of the paw withdrawal thresholds on the ipsilateral (ipsi) and contralateral (contra) sides. ***p < 0.001 compared with the threshold on day 0. ###*p *< 0.001 compared with the threshold of the vehicle-treated group. (B, C) Immunohistochemical (B) and western blot (C) analyses of p-cPLA_2 _protein in the ipsilateral L5 DRG of vehicle- and SB203580-treated rats 7 days after nerve injury. Scale bar, 50 μm.

An injury to the L5 nerve caused an increase in the phosphorylation of p38 mainly in small-diameter DRG neurons (data not shown), as previously demonstrated [22]. Nerve injury also induced an increase in ERK phosphorylation in satellite glial cells, and to a lesser extent, in large-diameter DRG neurons (Figure 1B). To examine the involvement of ERK and p38 in cPLA_2 _activation in DRG neurons, we tested the effects of inhibitors for MAPK kinase (MEK, a kinase upstream of ERK) and p38 that were administered through a catheter whose tip was positioned near the L5 DRG [2]. Vehicle-treated rats with an L5 nerve injury displayed a marked decrease in paw withdrawal threshold after nerve injury (Figure 1A and Figure 2A). By contrast, U0126, a selective inhibitor of MEK, and SB203580, a potent inhibitor of p38, significantly suppressed the development of tactile allodynia, as previously demonstrated [22] (U0126, *p *< 0.001; SB203580, *p *< 0.001) (Figure 1A and Figure 2A). Whereas p38 phosphorylation in DRG neurons was not inhibited by SB203580 (data not shown), because SB203580 binds to the ATP pocket in p38 to inhibit its kinase activity [32], ERK phosphorylation was suppressed by U0126 (Figure 1B). However, on day 7, the levels of phosphorylated cPLA_2 _(p-cPLA_2_) in the ipsilateral DRGs of U0126- and SB203580-treated rats were not changed compared with that in vehicle-treated rats. (Figure 1B and Figure 1C, Figure 2B and Figure 2C). Similar results were obtained in immunohistochemical analyses using the ipsilateral L5 DRG that was removed 45–60 min after injection of these inhibitors (data not shown). These results indicate that p38 and ERK are not involved in nerve injury-induced cPLA_2 _activation in DRG neurons.

### Peripheral nerve injury induces CaMKII activation in primary afferent neurons

**Figure 3:**
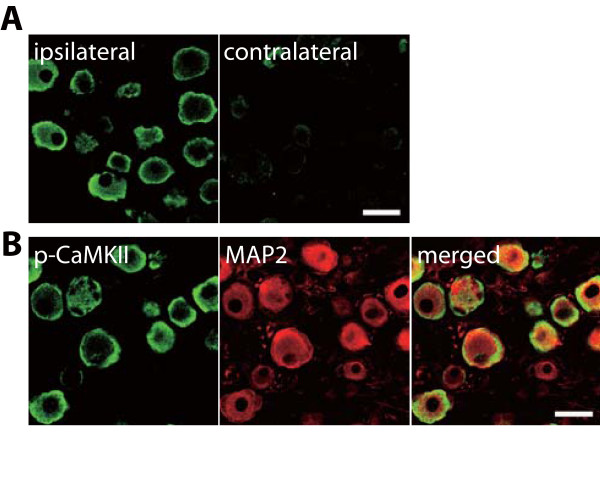
**CaMKII is activated in DRG neurons after nerve injury**. (A) Immunohistochemical analysis of p-CaMKII protein in the L5 DRG 7 days after nerve injury. (B) Double immunofluorescence labeling of p-CaMKII with MAP2, a marker of neurons, in the ipsilateral L5 DRG 7 days after nerve injury. Scale bar, 50 μm.

To examine whether spinal nerve injury induces the activation of CaMKII in DRG neurons, we performed immunohistochemical analysis using the DRGs of nerve-injured rats. We found that L5 nerve injury caused an increase in the level of phosphorylated CaMKII-immunoreactivity (p-CaMKII-IR) in the ipsilateral L5 DRG (Figure 3A). Accumulated p-CaMKII-IR was not observed in the contralateral DRG (Figure 3A). At the subcellular level, p-CaMKII-IR in damaged DRG neurons was accumulated at the edges of the area immunostained with the neuronal marker microtubule-associated protein 2 (MAP2) (Figure 3B).

### A CaMKII inhibitor suppresses nerve injury-induced cPLA_2 _activation

**Figure 4:**
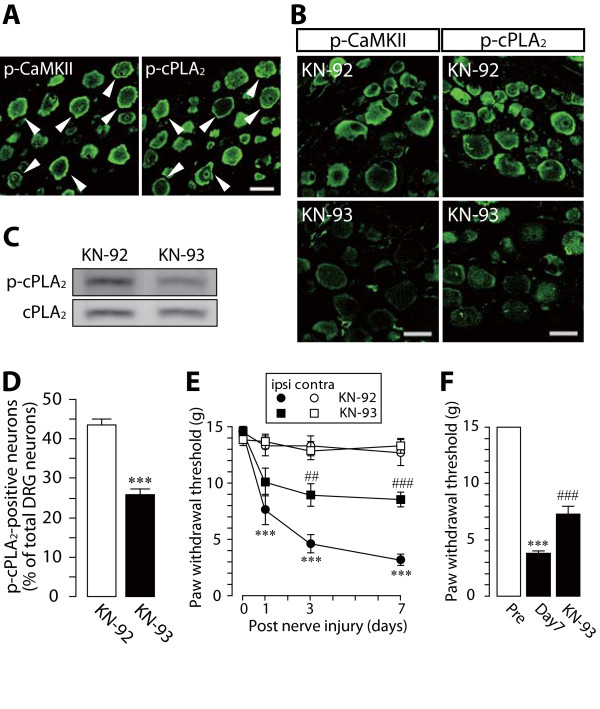
**A CaMKII inhibitor KN-93 suppresses tactile allodynia and cPLA_2 _activation**. (A-C) Immunohistochemical (A, B) and western blot (C) analyses of p-CaMKII and p-cPLA_2 _proteins in the ipsilateral L5 DRG of KN-92- and KN-93-treated rats 7 days after nerve injury. Arrowheads show DRG neurons positive for both p-CaMKII and p-cPLA_2_. Scale bar, 50 μm. (D) The number of p-cPLA_2_-translocated neurons in the ipsilateral L5 DRG 7 days after nerve injury. About 1000 neurons profiles were counted in twenty randomly chosen sections from six rats in each group. Results are percentages (means ± SEM) of p-cPLA_2_-translocated neurons (p-cPLA_2 _neurons) relative to the total number of neurons. ***p < 0.001 compared with the KN-92-treated group. (E) Effect of KN-93 on the development of nerve injury-induced tactile allodynia. Results are means ± SEM of the paw withdrawal thresholds on the ipsilateral (ipsi) and contralateral (contra) sides. ***p < 0.001 compared with the threshold on day 0. ##*p *< 0.01, ###*p *< 0.001 compared with the threshold of the KN-92-treated group. (F) Effect of a single administration of KN-93 on the decrease in paw withdrawal threshold (mean ± SEM) 7 days after nerve injury. ***p < 0.001 compared with pre-injury baseline (Pre). ###*p *< 0.001 compared with the threshold on day 7.

Recent evidence has indicated that CaMKII plays an important role in the phosphorylation of cPLA_2 _*in vitro *[14,21], suggesting a role for CaMKII in cPLA_2 _phosphorylation in injured DRG neurons. To investigate this hypothesis, we examined co-localization of phosphorylated cPLA_2 _and CaMKII in DRG neurons. Since it is difficult to perform double-immunolabeling of tissue with p-cPLA_2 _and p-CaMKII antibodies, because they were raised in the same host species (rabbit), we used two adjacent DRG sections and singly immunostained one section with each antibody. We observed DRG neurons that were positive for both p-cPLA_2 _and p-CaMKII in the injured DRG (indicated by arrowheads, Figure 4A). To further test whether the inhibition of CaMKII activation affects nerve injury-induced cPLA_2 _activation, we injected a CaMKII inhibitor, KN-93, and a negative control for KN-93, KN-92, into nerve-injured rats. We found that the levels of both p-CaMKII and p-cPLA_2 _in the ipsilateral DRG of KN-93-treated rats were much lower than those in KN-92-treated rats (Figure 4B and Figure 4C), and administration of KN-93 markedly reduced the number of DRG neurons showing translocated p-cPLA_2 _in response to nerve injury (*p *< 0.001) on day 7 (Figure 4D) compared with KN-92 (Figure 4D) or vehicle administration (*n *= 2, 38.3 ± 0.3%). These results suggest that CaMKII is involved in cPLA_2 _phosphorylation and translocation in DRG neurons caused by nerve injury. In addition, KN-93 significantly suppressed the development of tactile allodynia (day 3: *p *< 0.01, day 7: *p *< 0.001) (Figure 4E) and a single administration of KN-93 near the DRG 7 days after nerve injury also significantly suppressed the expression of tactile allodynia (*p *< 0.001) (Figure 4F). No alteration in motor behavior after KN-93 treatment was observed (data not shown). Taken together, these results indicate that inhibiting CaMKII activation prevents the development and expression of nerve injury-induced tactile allodynia.

### ATP receptor-dependent activation of CaMKII in cultured primary sensory neurons

**Figure 5:**
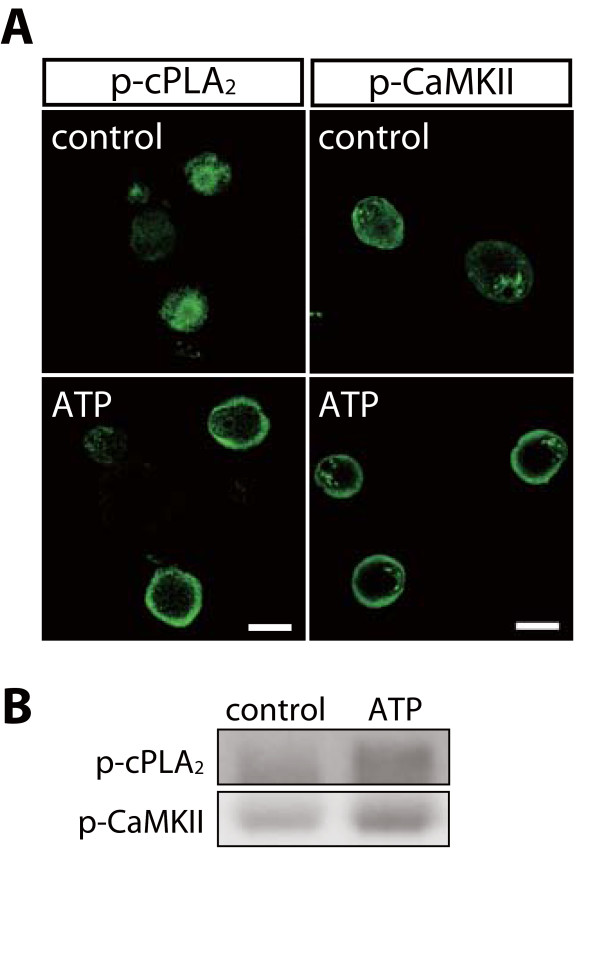
**cPLA_2 _and CaMKII are activated in the plasma membrane after applying ATP**. (A) Increase in the levels of p-cPLA_2 _and p-CaMKII immunofluorescence and translocation of these proteins to the plasma membrane in neurons 5 min after the application of 10 μM ATP compared with controls. Similar results were observed in each of three experiments. Scale bar, 20 μm. (B) The association between p-cPLA_2 _and p-CaMKII after application of 10 μM ATP. Protein samples were subjected to immunoprecipitation with the anti-p-cPLA_2 _antibody, and isolated complex was separated by electrophoresis and immunoblotted with anti-p-cPLA_2 _and anti-p-CaMKII antibodies.

**Figure 6:**
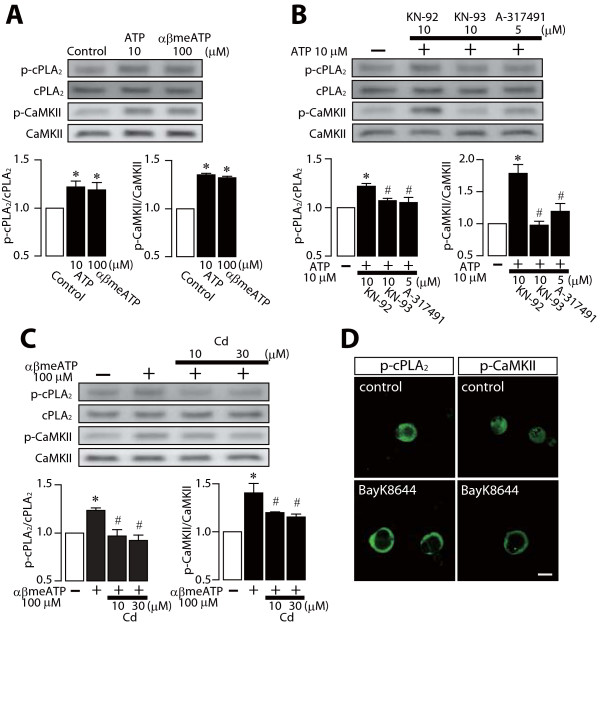
**cPLA_2 _and CaMKII are activated via stimulation of P2X_3 _and P2X_2+3 _receptors in primary DRG neurons**. (A-C) Western blot analyses of p-cPLA_2 _and p-CaMKII proteins after applying ATP or α,β-methylene ATP (αβmeATP) to rat primary DRG neurons. The primary DRG culture was incubated with ATP or αβmeATP. KN-92, KN-93, A-317491 or cadmium was added to the neurons 10 min before the application of ATP or αβmeATP. The total cPLA_2 _and CaMKII proteins loaded on each lane were also detected as loading controls. The bar graphs show the relative values of p-cPLA_2 _and p-CaMKII proteins induction with respect to controls, after normalizing for the cPLA_2 _and CaMKII protein levels, respectively. *p < 0.05 compared with the control group. #*p *< 0.05 compared with the ATP- or αβmeATP-stimulated group. (D) Increase in the levels of p-cPLA_2 _and p-CaMKII immunofluorescence and translocation of these proteins to the plasma membrane in neurons 5 min after the application of 10 μM BayK8644 compared with controls. Scale bar, 20 μm.

cPLA_2 _is activated via P2X_3_R/P2X_2+3_R in DRG neurons after nerve injury [2]. To examine whether CaMKII is also activated by these receptors, we applied ATP to cultured DRG neurons and found that the level of p-CaMKII-IR was increased in the vicinity of the plasma membranes of DRG neurons, similar to the translocation of p-cPLA_2 _after applying ATP (Figure 5A). Next, to investigate the physical association between p-CaMKII and p-cPLA_2_, we performed immunoprecipitation experiments and found that p-CaMKII was coimmunoprecipitated with p-cPLA_2 _in DRG neurons after the application of ATP (Figure 5B). Applying α,β-methylene ATP (αβmeATP), an agonist of P2X_1_- and P2X_3_-containing P2XRs, also increased p-cPLA_2 _and p-CaMKII levels (Figure 6A). When DRG neurons were pre-treated with KN-93 or A-317491, a potent and selective antagonist of P2X_3_R/P2X_2+3_R, prior to applying ATP, the ATP-induced phosphorylation of cPLA_2 _and CaMKII were significantly inhibited (Figure 6B). We also examined the contribution of voltage-dependent Ca^2+ ^channels (VDCCs) to CaMKII activation, because Ca^2+ ^influx via VDCCs has been reported to also be involved in CaMKII activation in neuronal cells [33]. The increases in the levels of p-cPLA_2 _and p-CaMKII induced by αβmeATP were abolished by cadmium, a nonselective blocker of VDCCs (Figure 6C). Furthermore, applying BayK8644, an agonist for VDCCs, to primary DRG neurons increased the level of p-CaMKII-IR in the vicinity of the plasma membranes of DRG neurons and caused the translocation of p-cPLA_2 _(Figure 6D). These results indicate that P2X_3_R/P2X_2+3_R and VDCCs have important roles in cPLA_2 _and CaMKII activation, and that p-cPLA_2 _is translocated to the plasma membranes of DRG neurons as a result of its interaction with p-CaMKII.

## Discussion

In the present study, we demonstrated for the first time that activation of CaMKII in DRG neurons is important for cPLA_2 _phosphorylation and translocation as well as the development and maintenance of neuropathic pain after peripheral nerve injury.

p38 and ERK have been shown to be involved in the phosphorylation of cPLA_2 _in non-neuronal cells [11-13] and to be activated in DRG neurons after spinal nerve injury [22]. Although p38 and MEK inhibitors administered near the injured DRG prevented the development of tactile allodynia, these did not affect the activation of cPLA_2_. It is thus conceivable that p38 and ERK in DRG neurons participate in the development of tactile allodynia through an independent pathway or downstream of the cPLA_2_-mediated signaling pathway. In particular, ERK and cPLA_2 _are activated in different types of cells in the injured DRG: ERK phosphorylation was seen predominantly in satellite glial cells, while activation of cPLA_2 _is observed mainly in medium-to-large-diameter DRG neurons [2], which supports our hypothesis. Alternatively, it is also possible that cPLA_2_-mediated lipid mediators, such as prostaglandins, platelet-activating factor and lysophosphatidic acid, which have been reported to produce tactile allodynia [6-9], may affect the excitation of DRG neurons via MAPK activation in primary afferent neurons. Studies in our laboratory determining cPLA_2_-mediated products and their roles in neuropathic pain are currently underway.

Recently, it was reported that cPLA_2 _is phosphorylated by CaMKII *in vitro *[14,21]. CaMKII is preferentially localized in pain-processing regions in the nervous system, such as the superficial laminae of the dorsal horn in the spinal cord and dorsal root ganglion [23,24]. CaMKII activity is significantly increased in the spinal cord after injection of capsaicin and formalin [34,35] and CFA-induced pain behaviors and increase of CaMKII phosphorylation in the spinal dorsal horn are reduced by KN-93 [36]. Furthermore, intrathecal injection of KN-93 attenuates the development of thermal hyperalgesia and mechanical allodynia following chronic constriction injury (CCI) [37]. These findings suggest that CaMKII expressed in the spinal cord contributes to chronic inflammatory and neuropathic pain as well as acute pain. By contrast, in DRG neurons, the phosphorylation of CaMKII has an important role in nerve growth factor-induced sensitization of TRPV1 [25] and modulation of the agonist binding to TRPV1 [26]. CaMKII expression in sensory neurons has been shown to be increased during chronic inflammation pain [24], but there have been no reports investigating the role of CaMKII in DRG neurons in neuropathic pain. In our immunohistochemical analyses, the level of CaMKII phosphorylation was increased in the ipsilateral DRG neurons after nerve injury. We also found that DRG neurons showing translocation of both phosphorylated cPLA_2 _and CaMKII to the plasma membrane were observed in the injured DRG. Importantly, pharmacological blockade of CaMKII prevented cPLA_2 _phosphorylation and translocation as well as tactile allodynia following peripheral nerve injury. These results suggest that the phosphorylation and translocation of cPLA_2 _to the plasma membrane via an interaction with activated CaMKII is a key event in the development of nerve injury-induced tactile allodynia. Our present behavioral study also reveals that KN-93 is effective in treating existing tactile allodynia, which is consistent with the behavioral analysis using a cPLA_2 _inhibitor [2]. Considering a previous result showing that KN-93 administered near the spinal cord 7 days after CCI produces no significant effect on existing tactile allodynia [37], the role of CaMKII in the maintenance phase of neuropathic pain may be predominant in the DRG rather than in the spinal cord.

Applying ATP caused an increase in the levels of both phosphorylated cPLA_2 _and CaMKII in the vicinity of the plasma membrane, and physical association of these two proteins in primary cultured DRG neurons. ATP receptor agonist-dependent phosphorylation of cPLA_2 _and CaMKII were inhibited either by the selective P2X_3_R/P2X_2+3_R antagonist A-317491 or by the nonselective VDCC blocker cadmium. Because the ATP-evoked current is not blocked by cadmium [38,39], our results suggest that Ca^2+ ^influx via the activation of P2X_3_R/P2X_2+3_R may not be enough to activate CaMKII and that VDCC activation (presumably resulting from a depolarization of DRG neurons by stimulating P2X_3_R/P2X_2+3_R) may contribute to CaMKII activation in DRG neurons. Activation of CaMKII and cPLA_2 _in A23187-stimulated DRG neurons supports this notion. Subsequently, activated CaMKII would phosphorylate cPLA_2 _and be translocated to the plasma membrane by interacting physically with activated cPLA_2_. To date, there have been many studies investigating CaMKII translocation and its roles in synaptic transmission and plasticity in the central nervous system. In hippocampal neurons, CaMKII is activated by Ca^2+ ^influx through NMDA receptors and then translocated to postsynaptic density (PSD) [40] in parallel with sustained CaMKII activity owing to an interaction with the NMDA receptor subunit NR2B [41]. Activated CaMKII subsequently phosphorylates many PSD proteins, including AMPA receptors, and binds to NMDA receptor subunits, resulting in induction of long-term potentiation [42,43]. In relation to pain, a previous study has reported that inhibition of CaMKII activity blocks translocation of AMPA receptor subunits to the plasma membrane of spinal cord neurons after capsaicin stimulation [44]. Although there have been few reports investigating the translocation of CaMKII in the peripheral nervous system, a recent study demonstrated that CaMKII activated by electric stimulation of the sciatic nerve is implicated in the trafficking of P2X_3_R toward the plasma membranes of DRG neurons [45]. Given the present data showing that cPLA_2 _and CaMKII are activated via stimulation of P2X_3_R/P2X_2+3_R in DRG neurons, this work indicates that P2X_3_R/P2X_2+3_R-dependent activation of cPLA_2 _and CaMKII is enhanced under pathological conditions, such as neuropathic pain.

## Conclusion

The present study demonstrated that CaMKII, but not MAPKs, has an important role in cPLA_2_-dependent tactile allodynia via the regulation of phosphorylation and translocation of cPLA_2_, both of which are mediated by P2X_3_R/P2X_2+3_R and voltage-dependent Ca^2+ ^channels in primary afferent neurons following peripheral nerve injury. Our results provide important evidence to help us to understand the mechanism underlying neuropathic pain modulated by cPLA_2 _and the translocation of cPLA_2 _and CaMKII in DRG neurons under pathophysiological conditions.

## Methods

### Animals

Male Wistar rats (250–300 g) were used. Animals were housed at a temperature of 22 ± 1°C with a 12-h light-dark cycle (light on 8:30 to 20:30), and fed food and water ad libitum. All of the animals used in the present study were obtained, housed, cared for and used in accordance with the guidelines of Kyushu University.

### Neuropathic pain model

We used the spinal nerve injury model [46] with some modifications [47]: in male Wistar rats a unilateral L5 spinal nerve was tightly ligated and cut just distal to the ligature. The mechanical allodynia was assessed by using calibrated von Frey filaments (0.4–15.1 g, Stoelting, Wood Dale, Illinois, USA) and the paw withdrawal threshold was determined as described previously [47].

### Drug treatment

Rats were implanted with catheters for intrathecal injection according to the method described previously. Under isoflurane anesthesia, a sterile 32 gauge intrathecal catheter (ReCathCo, Allison Park, Pennsylvannia, USA) was inserted through the atlanto-occipital membrane and to the L4 or L5 DRG and externalized through the skin [2]. After the experiments, we confirmed that the tip of the catheter was positioned near the L5 DRG. Rats were injected intrathecally with each drug using a 25 μl Hamilton syringe with a 30-gauge needle once a day from day 0 (just before the nerve injury) to day 6. The drugs used in this study are listed below: SB203580 (30 nmol/10 μl, Calbiochem, San Diego, California, USA), U0126 (10 nmol/10 μl, Promega, Madison, Wisconsin, USA), KN-92 (10 nmol/10 μl, Calbiochem) and KN-93 (10 nmol/10 μl, Calbiochem). The paw withdrawal threshold was tested 21–24 hr after the injection of each drug at 1, 3, 7 days post-injury. After the test on day 7, to examine the level of p-cPLA_2 _in injured DRG neurons in vehicle- and inhibitor-treated groups using immunohistochemistry and western blotting, the L5 DRG ipsilateral to the nerve injury was removed. For the experiment in which the effect of a single administration of KN-93 on the established allodynia was examined on day 7 after nerve injury, behavioral test was performed immediately before and after the injection of KN-93 (10 nmol/10 μl).

### Immunohistochemistry

Rats were deeply anesthetized by pentobarbital (100 mg/kg, i.p.) and perfused transcardially with 4% paraformaldehyde. DRG sections were removed, postfixed with the same fixative, and placed in 30% sucrose solution for 24 hr at 4°C. The DRG sections (15 μm) were incubated in a blocking solution [3% normal goat serum/0.3% Triton X-100/phosphate-buffered saline (PBS) (-) ] and then with anti-phospho-ERK (anti-p-ERK) antibody (1:500, Cell Signaling, Beverly, Massachusetts, USA), anti-phospho-cPLA_2 _(anti-p-cPLA_2_) antibody (1:200, Abcam, Cambridge, Massachusetts, USA) or anti-phospho-CaMKII (anti-p-CaMKII) antibody (1:500, Promega). Identification of the type of p-CaMKII-translocated cells was performed with MAP2, a marker of neurons (1:1000, Chemicon, Temecula, California, USA). Following incubation, the DRG sections were incubated with anti-rabbit immunoglobulin G (IgG)-conjugated Alexa Fluor 488 or anti-mouse IgG-conjugated Alexa Fluor 546 (1:1000, Molecular Probes, Eugene, Oregon, USA). The sections were then analyzed by a confocal microscope (LSM510, Zeiss, Oberkochen, Germany). The number of p-cPLA_2_-IR DRG neurons with translocation was counted in the L5 DRG ipsilateral to the nerve injury. The proportion of the p-cPLA_2_-translocated neurons to the total number of DRG neurons was determined in twenty randomly chosen sections from six rats in KN-92- and KN-93-treated groups.

### Western blotting

Rats were deeply anesthetized by pentobarbital (100 mg/kg, i.p.) and the L5 DRG ipsilateral to the nerve injury was quickly removed. The tissue was then homogenized in homogenization buffer (20 mM Tris-Hcl pH 7.4, 2 mM EDTA, 0.5 mM EGTA, 0.32 M sucrose, protease and phosphatase inhibitors cocktails) for 10 s on ice and centrifuged at 1000 × *g *for 5 min at 4°C to remove cell debris. The supernatant was transferred to a new tube, mixed with Laemmli sample buffer (125 mM Tris-HCl pH 7.4, 20% glycerol, 4% (w/v) sodium dodecyl sulfate (SDS), 0.025% (w/v) bromophenol blue and 5% 2-mercaptoethanol), and boiled at 95°C for 5 min. All samples were subjected to BCA assay to adjust the loading protein amount before adding the Laemmli sample buffer. The samples were subjected to a 7.5% polyacrylamide gel (BioRad, Hercules, CA, USA), and the proteins were transferred electrophoretically to polyvinylidene difluoride membranes. After blocking, the membranes were incubated with anti-p-cPLA_2 _antibody (1:1000, Cell Signaling), anti-cPLA_2 _antibody (1:1000, Cell Signaling) overnight at 4°C and then were incubated with horseradish peroxidase-conjugated anti-rabbit IgG antibody (1:1000, Amersham Biosciences, Buckinghamshire, UK). The blots were detected using a chemiluminescence method (ECL system; Amersham Biosciences).

### Culture of rat primary DRG neurons

The lumber DRGs (L1-6 segments) were removed from male Wistar rats and were treated in Dulbecco's modified eagle medium with 20 U/ml papain and 2 mg/ml collagenase type II for 1 hr at 37°C. At the end of this treatment the enzyme solution was removed and the DRGs were mechanically dissociated by trituration through a Pasteur pipette in Dulbecco's modified eagle medium. They were suspended in F-12 Nutrient Mixture liquid supplemented with 10% horse serum, 2 mM glutamine, 100 units/ml penicillin, 100 μg/ml streptomycin, 100 ng/ml nerve growth factor and 100 ng/ml human glial cell-line derived neurotrophic factor. They were plated in slide glasses or tissue culture dishes coated with 100 μg/ml poly-L-lysine and 10 μg/ml laminin and maintained in an atmosphere of 5% CO_2_/95% ambient air at 37°C for 72 hr. Following incubation, the medium was removed, replaced with fresh medium without horse serum, nerve growth factor and human glial cell-line derived neurotrophic factor, and further cultured at 37°C for an additional 24 hr.

DRG neurons were incubated with ATP for 5 min, and KN-92, KN-93, A-317491 (Sigma, St Louis, MO, USA) or cadmium (Sigma) was added to the neurons 10 min before the application of ATP. After these treatments, the medium was removed and the cultures were scraped into RIPA buffer (50 mM Tris-HCl (pH 7.4), 150 mM NaCl, 1% NP-40, 0.1% (w/v) SDS, 0.5% deoxycholate, protease and phosphatase inhibitors cocktails) and centrifuged at 21,600 × *g *for 30 min at 4°C to remove cell debris. The supernatant was transferred to a new tube, mixed with Laemmli sample buffer and boiled at 95°C for 5 min. Western blotting was carried out as described above. We used anti-p-CaMKII antibody (1:1000, Promega) or anti-CaMKII antibody (1:1000, Calbiochem) as additional primary antibodies.

Immunocytochemistry was performed as follows. Immediately after treatment with ATP or BayK8644 (Sigma) for 5 min, cells were fixed with 3.7% formaldehyde. After blocking, neurons were incubated with anti-p-cPLA_2 _antibody (1:200, Abcam) or anti-p-CaMKII antibody (1:500, Promega) and then were incubated with anti-rabbit IgG-conjugated Alexa Fluor 488 (1:1000, Molecular Probes) followed by analysis with an LSM510 Imaging System (Zeiss).

### Immunoprecipitation

Immediately after treatment with ATP for 5 min, cultured DRG neurons were rinsed once with PBS (-). RIPA buffer was added to each plate and plates were incubated on ice for 30 min. The cultured cells were scraped off and sonicated on ice three times for 5 s each. Protein samples were centrifuged at 21,600 × *g *for 30 min at 4°C and then the supernatants were transferred to a new tube, preabsorbed with anti-rabbit IgG beads (eBioscience, San Diego, California, USA) for 3 hr. The precleared protein extracts were incubated with anti-p-cPLA_2 _antibody (1:50, Cell Signaling) overnight at 4°C. Anti-rabbit IgG beads were subsequently added to the samples, and the mixture was further incubated for 1hr at 4°C. The protein beads complexes were washed four times with lysis buffer (50 mM Tris-HCl pH 8.0, 150 mM NaCl, 1% NP-40), and proteins were eluted by boiling for 10 min in Laemmli buffer. Samples were probed by western blotting using the corresponding primary antibodies and Rabbit TrueBlot-horseradish peroxidase anti-rabbit IgG (1:1000, eBioscience) as a secondary antibody. The blots were detected using a chemiluminescence method (ECL system).

### Statistical analysis

All data are presented as means ± SEM. The statistical significance of difference between values was determined by Student's *t *test, Mann-Whitney *U *test or analysis of variance (ANOVA) with appropriate *post hoc *tests. A *p *value less than 0.05 was considered to be statistically significant.

## Competing interests

The authors declare that they have no competing interests.

## Authors' contributions

SH participated in the design of the study, carried out all experiments, performed the statistical analysis and wrote the manuscript. YK performed the part of the behavioral test and immunohistochemical staining. MT participated in designing the study and wrote the manuscript. KI supervised the experiments and wrote the manuscript. All authors read and approved the final manuscript.
